# Isoprenoids increase bovine endometrial stromal cell tolerance to the cholesterol-dependent cytolysin from *Trueperella pyogenes*†

**DOI:** 10.1093/biolre/ioy099

**Published:** 2018-04-24

**Authors:** Sholeem Griffin, Gareth D Healey, I Martin Sheldon

**Affiliations:** Swansea University Medical School, Swansea University, Swansea, UK

**Keywords:** cow, infection, immunology, metabolism, stroma, uterus

## Abstract

Preventing postpartum uterine disease depends on the ability of endometrial cells to tolerate the presence of the bacteria that invade the uterus after parturition. Postpartum uterine disease and endometrial pathology in cattle are most associated with the pathogen *Trueperella pyogenes. Trueperella pyogenes* secretes a cholesterol-dependent cytolysin, pyolysin, which causes cytolysis by forming pores in the plasma membrane of endometrial stromal cells. The aim of the present study was to identify cell-intrinsic pathways that increase bovine endometrial stromal cell tolerance to pyolysin. Pyolysin caused dose-dependent cytolysis of bovine endometrial stromal cells and leakage of lactate dehydrogenase into supernatants. Cell tolerance to pyolysin was increased by inhibitors that target the mevalonate and cholesterol synthesis pathway, but not the mitogen-activated protein kinase, cell cycle, or metabolic pathways. Cellular cholesterol was reduced and cell tolerance to pyolysin was increased by supplying the mevalonate-derived isoprenoid farnesyl pyrophosphate, or by inhibiting farnesyl-diphosphate farnesyltransferase 1 or geranylgeranyl diphosphate synthase 1 to increase the abundance of farnesyl pyrophosphate. Supplying the mevalonate-derived isoprenoid geranylgeranyl pyrophosphate also increased cell tolerance to pyolysin, but independent of changes in cellular cholesterol. However, geranylgeranyl pyrophosphate inhibits nuclear receptor subfamily 1 group H receptors (NR1H, also known as liver X receptors), and reducing the expression of the genes encoding NR1H3 or NR1H2 increased stromal cell tolerance to pyolysin. In conclusion, mevalonate-derived isoprenoids increased bovine endometrial stromal cell tolerance to pyolysin, which was associated with reducing cellular cholesterol and inhibiting NR1H receptors.

## Teaser

Mevalonate-derived isoprenoids, farnesyl and geranylgeranyl pyrophosphate, increase bovine endometrial stromal cell tolerance to the cholesterol-dependent cytolysin, pyolysin, which is secreted by the uterine pathogen *Trueperella pyogenes.*

## Introduction

Bacteria are ubiquitous in the uterus of dairy cattle after parturition, and infections of the endometrium cause metritis or endometritis in 20% to 40% of animals [1–3]. The cost of treating metritis, the subsequent infertility, and reduced milk production is estimated to be about $2 billion per annum to the dairy industries of the EU and USA [1]. Postpartum uterine disease and endometrial pathology are most associated with the pathogen *Trueperella pyogenes*, which secretes a virulence factor, pyolysin, that forms pores in the plasma membrane of cells [4–6]. Endometrial stromal cells are particularly susceptible to cytolysis caused by pyolysin, and disruption of the epithelium after parturition exposes the underlying sensitive stroma to pyolysin [5, 7]. The defensive capability of a host against pathogens depends on resistance and tolerance [8, 9]. Resistance is the ability to reduce the pathogen burden, usually by immune cells killing the microbes. However, pyolysin does not provoke innate immune responses in endometrial or immune cells [5]. Tolerance is the ability to limit the impact of pathogens on health by tolerating a given microbial burden. Although tolerance to pathogens and their virulence factors, such as pyolysin, is thought to be vital for maintaining animal health, very few host-encoded tolerance mechanisms have been identified in any animal species [10].

**Figure 1.**
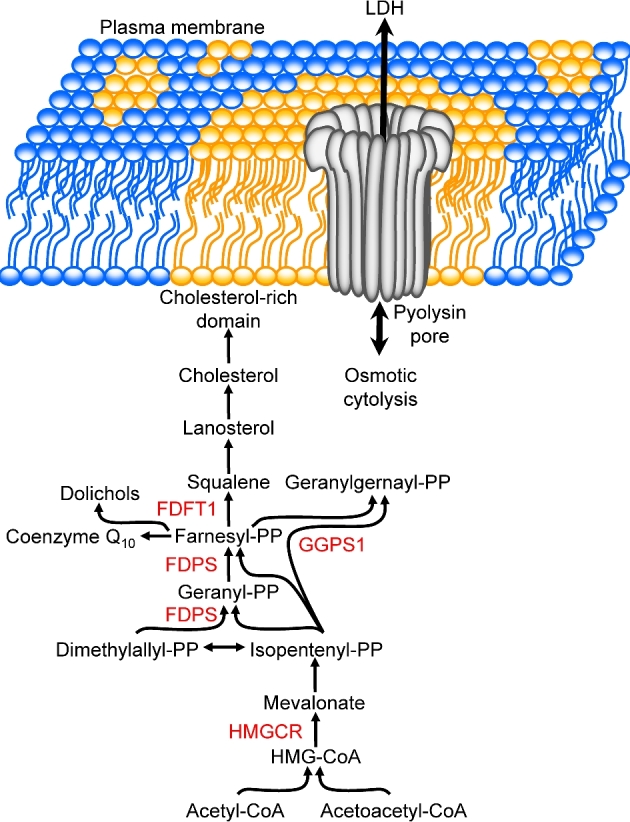
Cholesterol-dependent cytolysin. Schematic illustration of a pore formed by pyolysin in a cholesterol-rich domain in the plasma membrane, allowing leakage of ions and cytosolic molecules, such as lactate dehydrogenase (LDH). Cholesterol is derived from the mevalonate pathway that initially leads to the synthesis of the isoprenoid farnesyl pyrophosphate, with key enzymes indicated for 3-hydroxy-3-methylglutaryl-coenzyme A reductase (HMGCR) and farnesyl diphosphate synthase (FDPS). Conversion of farnesyl pyrophosphate to squalene by farnesyl-diphosphate farnesyltransferase 1 (FDFT1) is the first committed step of cholesterol synthesis, whilst geranylgeranyl diphosphate synthase 1 (GGPS1) yields the isoprenoid geranylgeranyl pyrophosphate. PP, pyrophosphate.

After parturition, the uterus of dairy cattle contains a range of bacteria, including *T. pyogenes*, which come from the environment and the uterine microflora [1, 11–13]. *Trueperella pyogenes* is important because it is associated with the clinical severity of uterine disease and the extent of the subsequent infertility [1, 4, 13]. Furthermore, only the presence of *T. pyogenes* is correlated with the severity of endometrial pathology [4, 6]. Pyolysin (also known as PLO) is the major virulence factor of *T. pyogenes*, and pyolysin is a member of the cholesterol-dependent cytolysin family of pore-forming toxins [5, 14, 15]. Cholesterol-dependent cytolysins are secreted from bacteria in a water-soluble form but convert into multimers in cholesterol-rich domains of the plasma membrane of mammalian cells, where they create 30 to 50 nm diameter transmembrane pores (Figure 1) [16–18]. These pores allow leakage of ions and cellular proteins across the plasma membrane, leading to cytolysis [16, 17, 19]. Bovine endometrial stromal cells are more sensitive to cytolysis caused by pyolysin than endometrial epithelial cells, neutrophils, monocytes, or lymphocytes [5, 7].

Tolerance to pyolysin depends on the resilience of stromal cells to endure or cope with damage during infections. As pyolysin forms pores in the plasma membrane of cells, we reasoned that cell-intrinsic pathways might regulate the ability of cells to tolerate pyolysin. Plasma membranes contain 90% cellular cholesterol, and cholesterol constitutes almost half of the lipid molecules [20]. Cholesterol is synthesized by a series of enzymes, with the mevalonate pathway initially condensing two acetyl-CoA molecules to form acetoacetyl-CoA, which is then converted to 3-hydroxy-3-methyl-glutaryl-CoA (HMG-CoA) by 3-hydroxy-3-methylglutaryl-CoA synthase 1, before 3-hydroxy-3-methylglutaryl-CoA reductase (HMGCR) yields mevalonate [21, 22]. A series of steps convert mevalonate to isoprenoids, with farnesyl diphosphate synthase (FDPS) yielding farnesyl pyrophosphate (Figure 1). Farnesyl pyrophosphate is the key pathway branch point because it is a substrate for several enzymes, including farnesyl-diphosphate farnesyltransferase 1 (FDFT1, also known as squalene synthase) generating squalene, and geranylgeranyl diphosphate synthase 1 (GGPS1) forming geranylgeranyl pyrophosphate (Figure 1). Squalene is converted to lanosterol by lanosterol synthase, and a series of enzymes ultimately lead to the synthesis of cholesterol [23]. Cholesterol homeostasis and the mevalonate pathway are regulated by sterols, isoprenoids, and oxysterols, and their intracellular sensors [24, 25]. For example, cholesterol-derived oxysterols and geranylgeranyl pyrophosphate bind the intracellular nuclear receptor subfamily 1 group H member 3 and 2 receptors (NR1H3 and NR1H2; also known as liver X receptors, LXRα and LXRβ, respectively) to regulate the expression of HMGCR, FDPS, FDFT1, and ABCA1 (ATP binding cassette subfamily A member 1) [26–29]. Another cellular pathways that might be important for cell tolerance to pyolysin is the mitogen-activated protein kinase (MAPK) pathway, as some pore-forming toxins activate the cell stress molecules MAPK3/1, MAPK14, and MAPK8 (also known as ERK1/2, p38, and JNK, respectively) [30, 31]. Other important cell-intrinsic pathways, such as cell cycle regulators and the metabolic sensors, also influence plasma membrane turnover and cell viability [32–35]. Indeed, the risk of developing uterine disease is increased by the metabolic stress associated with milk production [36, 37].

The aim of the present study was to identify cell-intrinsic pathways that increase bovine endometrial stromal cell tolerance to pyolysin. We screened molecules that modulate the MAPK, cell cycle, metabolism, and cholesterol synthesis pathways. Cell tolerance to pyolysin was increased by manipulating the cholesterol synthesis pathway, and particularly by the mevalonate-derived isoprenoids farnesyl pyrophosphate and geranylgeranyl pyrophosphate. Changes in endometrial stromal cell tolerance to pyolysin were associated with cholesterol and NR1H receptor-dependent mechanisms.

## Methods

### Pyolysin

The pyolysin plasmid (pGS59) was a generous gift from Prof. B.H. Jost (University of Arizona), and pyolysin protein was generated as described previously [5, 15]. The specific activity of pyolysin was 628,338 HU/mg protein, as determined using a hemolysis assay. There was very little endotoxin contamination (1.5 EU/mg protein), as determined by a limulus amebocyte lysate assay (LAL endotoxin quantitation kit; Thermo Scientific, Hertfordshire, UK).

### Hemolysis assay

To determine the activity of pyolysin, a hemolysis assay was performed using a 0.5% (v/v) suspension of horse red blood cells (Oxoid, Hampshire, UK) as described previously [5]. Optical density (OD_450_) was measured using a microplate reader (POLARstar Omega; BMG Labtech, Offenburg, Germany). Hemolytic units were mathematically determined by four-parameter modeling using the Solver function in Microsoft Excel.

To examine for potential binding of pyolysin to farnesyl pyrophosphate or geranylgeranyl pyrophosphate, 100 HU/ml pyolysin was incubated for 1 h with 20 μM farnesyl pyrophosphate or 20 μM geranylgeranyl pyrophosphate, using 1 mM cholesterol as a positive control because cholesterol binds pyolysin [19]. A hemolysis assay was conducted after the 1-h incubation, as described above.

### Cell culture

Isolation and culture of primary bovine endometrial stromal cells was performed as described previously [38, 39]. Briefly, uteri with no gross evidence of genital disease or microbial infections were collected from cattle after they were slaughtered and processed as part of the normal work of an abattoir. Postpartum cattle were not used to isolate endometrial cells because experiments would be confounded by the ubiquitous bacterial contamination of the uterus after parturition, existing endometrial inflammation and damage, and the metabolic stress of lactation [36–40]. Stromal cells were isolated by enzymatic digestion of the endometrium, sieving the cell suspension through a 40-μm mesh, and then adhesion of the stromal cells to culture plates, as described previously [38, 39]. Stromal cells were maintained at 37°C in humidified air with 5% carbon dioxide, using complete culture medium comprising RPMI-1640 (Gibco, Gaithersburg, MD) with 10% fetal bovine serum (Biosera, Heathfield, UK), and 50 IU/ml penicillin, 50 μg/ml streptomycin, and 2.5 μg/ml amphotericin B (all Sigma, Gillingham, UK).

### Cell experiments

Stromal cells were seeded at a density of 50,000 cells per well in 24-well tissue culture plates (TPP, Trasadingen, Switzerland), and cultured in complete culture medium using 1 ml/well to approximately 70% confluence, as described previously [19, 39]. To avoid confounding cell-extrinsic factors in serum that might affect intracellular signaling pathways and cholesterol homeostasis, cells were then cultured in serum-free culture medium and treatments (see below) were applied in serum-free culture medium comprising RPMI-1640 with 50 IU/ml penicillin, 50 μg/ml streptomycin, and 2.5 μg/ml amphotericin B, as described previously [5, 19].

To screen for molecules and pathways that might increase cell survival when cells were challenged with pyolysin, stromal cells were incubated for 24 h in serum-free medium containing vehicle, or molecules that target: MAPK (5 μM ERK Activation Inhibitor Peptide I (Calbiochem 328000) to inhibit MAPK3/1, 10 μM JNK inhibitor II (Calbiochem 420128) to inhibit MAPK8, and 10 μM SB 203580 (Calbiochem 559398) to inhibit MAPK14, as used previously [38]); cell cycle (50 μM PNU112455A (Sigma SML0498) to inhibit cyclin dependent kinase (CDK)2 and CDK5, 80 μM roscovitine (Sigma R7772) to inhibit CDK1, CDK2, CDK5, and CDK7, and 3 μM butyrolactone I (Sigma B7930) to inhibit CDK1, CDK2, and CDK5 [35, 41]); metabolic signaling pathways (10 μM AICAR (Cell Signaling 9944) to activate AMPK, 100 μM compound C (Calbiochem 171261) to inhibit AMPK, 2 μM Torin 1 (Cell Signaling 14379) to inhibit mTOR, 4 μM rapamycin (Cell Signaling 9904) to inhibit mTOR, 40 μM AKT inhibitor IV (Calbiochem 124038), and 2.8 μM LY294002 (Cell Signaling 9901) to inhibit phosphoinositide 3-kinases [34, 42, 43]); or 48 h incubation with inhibitors for the cholesterol synthesis pathway (1 μM atorvastatin (Sigma PZ0001) to inhibit HMGCR [44]; 100 μM etidronate (Sigma P5248) and 10 μM alendronate (Sigma A4978), which inhibit human FDPS with IC_50_ of 80 and 0.5 μM, respectively [45]; 10 μM zaragozic acid (Sigma Z2626), which inhibits FDFT1 [19, 46]). Methyl-β-cyclodextrin (0.5 mM, Sigma C4555) was used as a positive control because this cyclic oligosaccharide has an internal cavity that encapsulates hydrophobic sterols to efficiently deplete cholesterol from cells, which protects cells against cholesterol-dependent cytolysins [5, 18, 47]. The duration of treatment was based on our previous studies and preliminary experiments [5, 19, 31, 48]. After 24-h treatment, the supernatants were discarded and the cells were challenged with control serum-free medium or medium containing 100 HU/ml pyolysin for 24 h, and cell viability was determined by MTT assay.

Having identified cholesterol synthesis as a target pathway for protecting cells against pyolysin, we sought to test cell tolerance using a brief 2-h challenge with pyolysin because cell viability after a 24-h challenge with pyolysin might reflect cell repair or replication as well as tolerance to pyolysin. To first determine the tolerance of stromal cells to pyolysin, and identify a suitable pyolysin challenge for subsequent experiments, cells were challenged for 2 h with control serum-free culture medium or medium containing a range of concentrations of pyolysin from 1 to 200 HU/ml, as indicated in the Results section. At the end of the 2-h challenge period, supernatants were collected for measurement of LDH leakage from cells, and cell viability was examined by MTT assay.

To then examine the effect of components of the cholesterol synthesis pathway and mevalonate-derived isoprenoids on cell tolerance to pyolysin, cells were treated for 48 h in serum-free culture medium or medium containing cell-soluble mevalonate (Sigma 90469), farnesyl pyrophosphate (Sigma F6892), squalene (Sigma S3626), lanosterol (Sigma L5768), geranylgeranyl pyrophosphate (Sigma G6025), or methyl-β-cyclodextrin (Sigma C4555), using the concentrations indicated in the Results section. Media were discarded and the cells were challenged for 2 h with control culture medium or medium containing 100 HU/ml pyolysin. Alternatively, cells were transfected with scramble siRNA (ON-TARGETplus Non-targeting siRNA #1; Dharmacon; gelifesciences.com) or siRNA targeting *FDPS, GGPS1, NR1H2*, or *NR1H3*, which were designed using Dharmacon siDESIGN Center (Supplemental Table S1). Briefly, duplex complexes were formed by adding 100 pM of siRNA to 500 μl/well Opti-MEM I medium (Invitrogen, Waltham, MA) in 6-well plates (TPP), and then adding 7.5 μl Lipofectamine RNAiMAX Reagent (Invitrogen). Following 20-min incubation at room temperature, 250,000 exponentially growing cells were then seeded in 2.5 ml/well in complete culture medium for 48 h, and then 24 h in serum-free culture medium, prior to challenge for 2 h with control serum-free culture medium or medium containing 100 HU/ml pyolysin, and then examining cell viability and the leakage of LDH from cells into supernatants.

### Cell viability and LDH assay

Cell viability was assessed by the mitochondria-dependent reduction of 3-(4,5-dimethylthiazol-2-yl)-2,5-diphenyltetrazolium bromide (MTT, Sigma) to formazan, as described previously [5, 49]. Briefly, once supernatants were collected, the remaining cells were incubated for 2 h in 250 μl/well serum-free culture medium containing 1 mg/ml MTT; the medium was then removed and the cells lysed with 250 μl/well dimethyl sulfoxide (Sigma), and optical density (OD_570_) measured using a POLARstar Omega microplate reader. We assumed that a reduction in OD_570_ was a reflection of cytolysis, as described previously for experiments using cholesterol-dependent cytolysins [5, 31, 50]. The correlation between MTT OD_570_ measurements and the number of live cells was confirmed previously using trypan blue exclusion and counting the number of live cells using a hemocytometer [5]. We defined 100% viability as the OD_570_ measurement for cells in control medium, and the percentage viability was calculated as the OD_570_ of pyolysin-treated cells relative to OD_570_ values for cells in control medium.

Leakage of cellular LDH was measured in cell culture supernatants using the Lactate Dehydrogenase Activity Colorimetric Assay Kit (Biovision, California), according to the manufacturer's instructions. We assumed that increased LDH concentrations were a reflection of pore formation, as described previously for experiments using cholesterol-dependent cytolysins [31, 51]. For our experiments, we defined 100% LDH leakage as the LDH concentration in the supernatants of cells challenged with 100 HU/ml pyolysin, and the percentage LDH leakage was calculated as the LDH abundance of treated cells challenged with pyolysin relative to untreated cells challenged with pyolysin.

### Cholesterol measurement

Cells were cultured to 70% confluence in 12-well tissue culture plates (TPP) and treated with mevalonate pathway intermediates or siRNA, as described above. At the end of the incubation period, cells were collected in 200 μl/well cholesterol assay buffer (Invitrogen) and stored in Eppendorf tubes at –20°C. Prior to cholesterol quantitation, samples were defrosted at room temperature and sonicated for 10 min in a sonicating water bath. Cellular cholesterol concentration was measured using the Amplex Red Cholesterol Assay Kit (Invitrogen), according to the manufacturer's instructions. To normalize the concentrations of cellular cholesterol, phospholipid was measured in the same samples using a phospholipid assay kit (Sigma Aldrich), according to the manufacturer's guidelines.

### Quantitative PCR

Cellular RNA was extracted using the RNeasy Mini Kit according to the manufacturer's instructions (Qiagen, Crawley, UK). The RNA was quantified using a Nanodrop ND1000 spectrophotometer (Labtech, Ringmer, UK), and 1 μg of total RNA was added to a genomic DNA elimination reaction, followed by conversion to cDNA (Quantitect Reverse Transcription Kit, Qiagen), according to the manufacturer's instructions. Quantitative PCR was performed using exon–exon junction spanning primers (Supplemental Table S2) and the IQ5 system (Bio-Rad, Hemel Hempstead, UK). The starting quantity of mRNA was determined using standard curves generated from serial dilutions of pooled reference RNA with Quantifast SYBR green (Qiagen). The target and reference genes were analyzed in triplicate, and mRNA expression normalized to the *ACTB* and *RPL19* reference genes (Supplemental Table S2) using the IQ5 system (Bio-Rad), with inter-run correlation and run-dependent differences corrected using qBase software on the IQ5 system (Bio-Rad), as described previously [52]. The reference genes did not differ in their expression with the treatments, and the reference genes were amplified with the same efficiency as the target genes.

### Western blotting

The abundance of ABCA1 protein was used to verify the effectiveness of siRNA targeting NR1H receptors [29]. Cells were stored in RIPA buffer at –80°C for western blotting. Cell lysate proteins were normalized to 1 μg/μl using the DC Assay (Bio-Rad) and separated (10 μg per lane) using 10% (vol/vol) SDS-polyacrylamide gel electrophoresis. Prestained molecular weight markers were run in parallel lanes (Bio-Rad). After electrophoresis, proteins were transferred to a polyvinylidene difluoride membrane (Bio-Rad); nonspecific sites were blocked using a solution of 5% (wt/vol) bovine serum albumin (Sigma) in Tris-buffered saline (TBS) overnight at 4°C with gentle agitation. Membranes were probed with antibodies targeting ABCA1 (Abcam Cat# ab18180, RRID:AB_444302; Abcam, Cambridge, UK), which was selected based on recognition of immunoreactive proteins of 254 kDa (Supplemental Table S3), and protein loading was evaluated and normalized by examining ACTB (actin beta; Abcam Cat# ab8226, RRID:AB_306371; Abcam). Primary antibodies were used at 1:500 dilutions in 5% (wt/vol) BSA in TBS for 2 h with gentle agitation. After incubation, membranes were washed three times for 5 min in TBS and 0.1% Tween 20 (pH 7.6). Membranes were then incubated in secondary horseradish peroxidase-conjugated antibody (Cell Signaling Technology, Danvers, MA) in 5% (wt/vol) BSA in TBS for 1.5 h, and washed three times for 5 min in TBS and 0.1% Tween 20 (pH 7.6). Steady-state levels of immunoreactive proteins were visualized using enhanced chemiluminescence (Western C; Bio-Rad). The average peak densities of unsaturated bands were analyzed using Quantity-one software (Bio-Rad), and normalized to ACTB abundance.

### Statistical analysis

Data are presented as arithmetic mean and error bars represent SEM. The statistical unit was each animal from which cells were isolated. Statistical analyses were performed using GraphPad Prism 5.0.1 and SPSS 20.0, with *P* < 0.05 considered statistically significant. Comparisons between two treatments were tested using the Student *t*-test, and amongst several treatments using ANOVA, followed by the Dunnett post hoc multiple comparison test.

## Results

### Endometrial stromal cell tolerance to pyolysin

**Figure 2.**
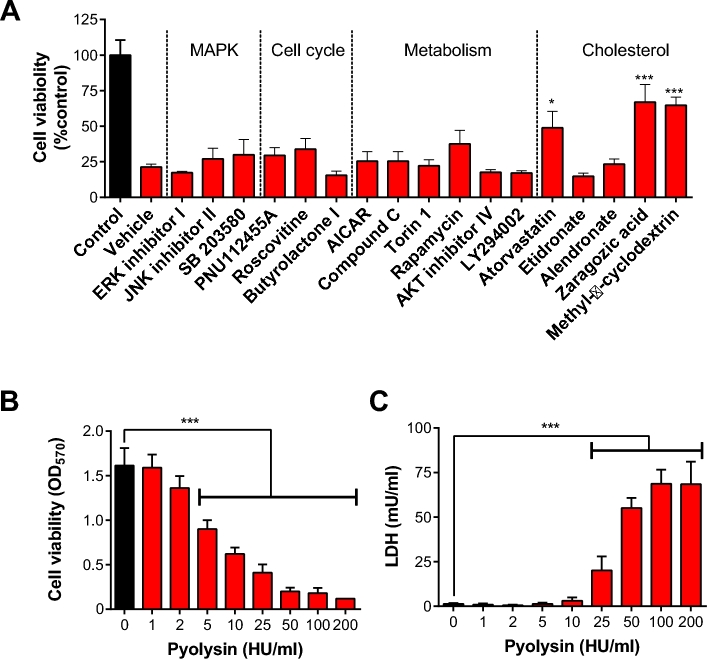
Cellular sensitivity to pyolysin. (A) Bovine endometrial stromal cells were incubated with culture medium containing vehicle, or molecules that target MAPK (5 μM ERK inhibitor, 10 μM JNK II inhibitor, 10 μM p38 inhibitor), cell cycle (50 μM PNU112455A, 80 μM roscovitine, 3 μM butyrolactone I), metabolic signaling pathways (10 μM AICAR, 100 μM compound C, 2 μM Torin 1, 4 μM rapamycin, 40 μM AKT inhibitor IV, 2.8 μM LY294002), or the cholesterol synthesis pathway (1 μM atorvastatin, 100 μM etidronate, 10 μM alendronate, 10 μM zaragozic acid), with 0.5 mM methyl-β-cyclodextrin as a positive control. Cells were then challenged with control medium, black bar (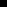) or medium containing 100 HU/ml pyolysin red bars (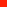), and cell viability was determined by MTT assay. Data are from at least three animals per treatment, and expressed as percent cell viability compared with cells in control medium. Data are presented as mean (SEM), and were analyzed by one-way ANOVA and Dunnett multiple comparison post hoc test; values differ from vehicle, * *P* < 0.05, *** *P* < 0.001. (B, C) Bovine endometrial stromal cells were incubated for 2 h with control medium black bar (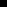) or medium containing the indicated concentrations of pyolysin red bars (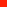). Cell viability was evaluated by MTT assay (B) and leakage of LDH into cell supernatants (C). Data are presented as mean (SEM); n = 4 animals. Data were analyzed by ANOVA with Dunnett multiple comparison test; values differ from control *** *P* < 0.001. (Please see the online version for the color figure.)

To initially screen for cellular pathways that may increase cell tolerance, stromal cells were treated with inhibitors or agonists that target the MAPK, cell cycle, metabolic and cholesterol pathways, prior to challenge with pyolysin. Treatments were prepared in serum-free culture medium using concentrations recommended by the manufacturers or in the literature. The MAPK and cholesterol pathways are established regulators of cell tolerance to cholesterol-dependent cytolysins [19, 30]. We considered cell cycle because cell replication requires plasma membrane turnover [35]. Furthermore, in preliminary experiments, 24-h treatment with a sublytic concentration of 0.25 HU/ml pyolysin, in serum-free medium, increased cell abundance (126 ± 5% of control, *P* < 0.05, *t*-test). The AMPK, mTOR, AKT, and PI3K metabolic signaling pathways are associated with postpartum metabolic stress, postpartum uterine disease, and changes in endometrial resistance to pathogens [32–34, 36, 37, 43]. The cholesterol synthesis pathway was targeted using atorvastatin to inhibit HMGCR, etidronate and alendronate to inhibit FDPS, and zaragozic acid to inhibit FDFT1 [19, 44–46]. Methyl-β-cyclodextrin was used as a control because it depletes bovine endometrial stromal cell cholesterol, and is an established mechanism to increase cell tolerance to pyolysin [5, 31]. The inhibitors did not significantly reduce stromal cell viability per se, except for roscovitine (53.6 ± 5.1% of control, *P* < 0.05, t-test) and Compound C (37.4 ± 1.6% of control, *P* < 0.01, t-test). The pyolysin challenge caused a 79% reduction in cell viability compared with cells in control medium, as determined by MTT assay (Figure 2A). There was no significant effect on the viability of cells challenged with pyolysin associated with prior treatment with inhibitors or agonists for the MAPK, cell cycle, or metabolic signaling pathways (Figure 2A). However, cell tolerance to pyolysin was increased by atorvastatin, zaragozic acid, and methyl-β-cyclodextrin. These results provide evidence that modulating the cholesterol synthesis pathway may improve the ability of cells to cope with pyolysin.

The 24-h challenge with pyolysin might allow time for cell repair and replication, as well as reflecting cell tolerance to pyolysin. To specifically examine cell tolerance, bovine endometrial stromal cells were challenged with a range of concentrations of pyolysin for 2 h. This 2-h pyolysin challenge caused dose-dependent cytolysis, as determined by MTT assay (*P* < 0.001, ANOVA; Figure 2B), and dose-dependent formation of pores in the plasma membrane, as determined by leakage of LDH into the cell supernatants (*P* < 0.001, ANOVA; Figure 2C). To seek cellular pathways that increase cell tolerance to pyolysin, in subsequent experiments we selected a 100 HU/ml pyolysin challenge because this caused a near maximal reduction in cell viability and leakage of LDH (Figure 2B and C).

### Cholesterol synthesis pathway

**Figure 3.**
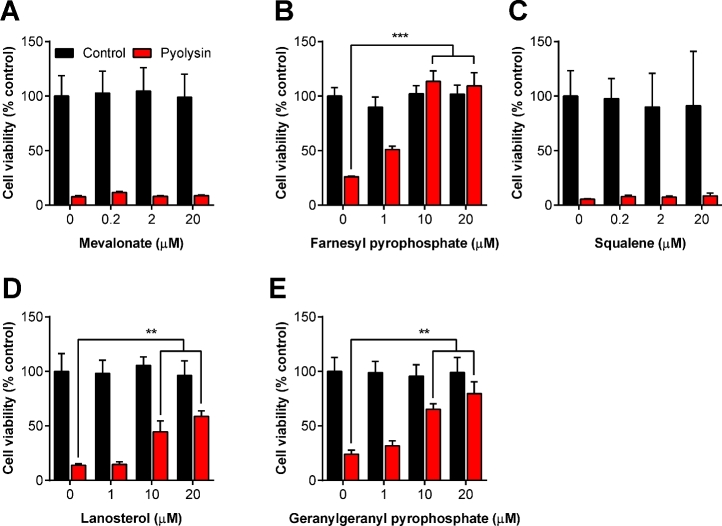
Farnesyl and geranylgeranyl pyrophosphate increase cell tolerance to pyolysin. Endometrial stromal cells were incubated with serum-free culture medium containing the indicated concentrations of (A) mevalonate, (B) farnesyl pyrophosphate, (C) squalene, (D) lanosterol, or (E) geranylgeranyl pyrophosphate for 48 h and then challenged with control serum-free medium black bars (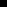) or medium containing 100 HU/ml pyolysin red bars (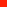) for 2 h. Cell viability was determined by MTT assay and data expressed as the percent of control. Data are presented as mean (SEM); n = 3 or 4 animals for each treatment. Data were analyzed by one-way ANOVA and Dunnett multiple comparison post hoc test; ** *P* < 0.01, *** *P* < 0.001. (Please see the online version for the color figure.)

To take another approach to exploring which parts of the cholesterol synthesis pathway might be important for cell tolerance, we supplemented endometrial stromal cells with cell-permeable intermediates of the cholesterol synthesis pathway (Figure 1). Cells were treated with mevalonate, farnesyl pyrophosphate, squalene, or lanosterol, prior to challenge with 100 HU/ml pyolysin (Figure 3A–D). Cytolysis was examined using the MTT assay, and cell viability was expressed as a percentage of the cells in control medium. Supplying mevalonate or squalene had no significant effect on cell tolerance to pyolysin (Figure 3A and C). However, supplying farnesyl pyrophosphate or lanosterol increased the tolerance of cells to pyolysin, with ≥10 μM farnesyl pyrophosphate providing complete tolerance to pyolysin (Figure 3B and D). The effect of farnesyl pyrophosphate was interesting as inhibiting FDFT1 with zaragozic acid also increases the abundance of farnesyl pyrophosphate [53], and zaragozic acid increased cell tolerance to pyolysin in Figure 1A. However, inhibiting FDFT1 also increases the abundance of geranylgeranyl-pyrophosphate [53], which is the isoprenoid derivate of farnesyl pyrophosphate (Figure 1). Treating stromal cells with geranylgeranyl pyrophosphate resulted in a concentration-dependent increase in cell tolerance to pyolysin (Figure 3E). One concern was that the cholesterol pathway intermediates added to cells might affect cell viability per se. However, there was no significant effect on endometrial stromal cell viability across the range of concentrations of mevalonate, farnesyl pyrophosphate, squalene, lanosterol, or geranylgeranyl pyrophosphate used in the present study (Figure 3A–E, black bars).

**Figure 4.**
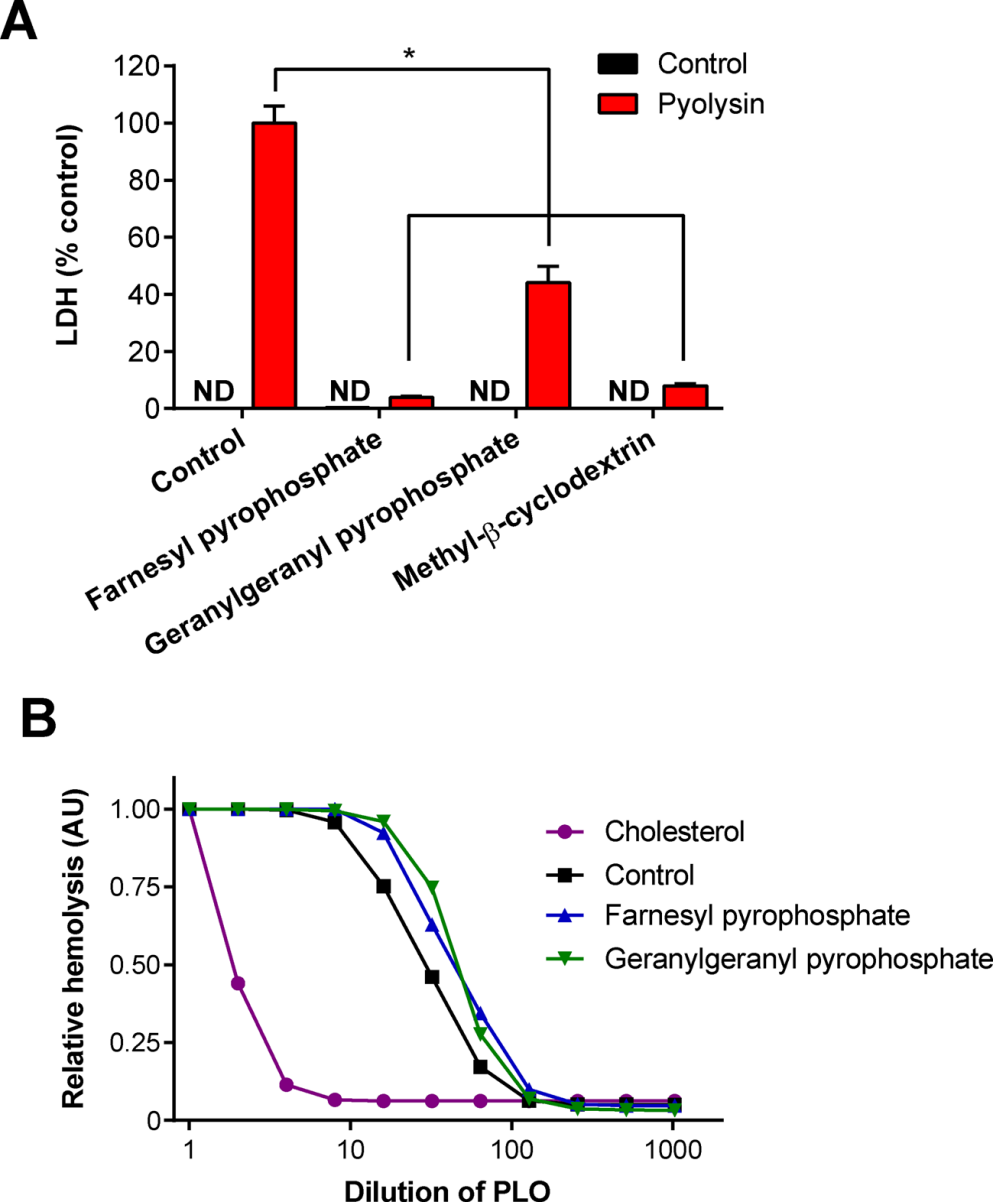
(A) Endometrial stromal cells were incubated in control serum-free medium, or medium containing 20 μM farnesyl pyrophosphate, 20 μM geranylgeranyl pyrophosphate, or 0.5 mM methyl-β-cyclodextrin, and then challenged with control serum-free medium black bars (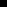) or medium containing 100 HU/ml pyolysin red bars (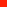) for 2 h. Cellular leakage was assessed by measuring LDH in cell supernatants, and data expressed as percent of control challenged with pyolysin. Data are presented as mean (SEM); n = 3 animals; ND, not detectable. Data were analyzed by one-way ANOVA and Dunnett multiple comparison post hoc test; values differ from pyolysin treatment, * *P* < 0.05. (B) Dulbecco PBS (control), 20 μM farnesyl pyrophosphate, 20 μM geranylgeranyl pyrophosphate, or 0.5 mM cholesterol as a positive control were incubated with the indicated concentrations of pyolysin for 1 h in DPBS, and hemolysis evaluated by further incubation with 0.5% (v/v) horse red blood cells for 1 h at 37°C. Data are presented as mean relative hemolysis in arbitrary units (AU); the group SEM was 0.13 AU; n = 3 independent experiments. (Please see the online version for the color figure.)

To further explore the effect of the isoprenoids on cell tolerance, the effect on leakage of LDH from cells was evaluated using stromal cells treated for 24 h with 20 μM farnesyl pyrophosphate or 20 μM geranylgeranyl pyrophosphate, prior to challenge with 100 HU/ml pyolysin. Treatment with farnesyl pyrophosphate or geranylgeranyl pyrophosphate reduced the leakage of LDH by 96% and 56%, respectively, compared with cells in control medium challenged with pyolysin (Figure 4A). As expected, treatment with 0.5 mM methyl-β-cyclodextrin, used as a positive control, also reduced LDH leakage (Figure 4A).

A potential confounding factor was that, although treatment media were removed prior to challenge with pyolysin, farnesyl pyrophosphate or geranylgeranyl pyrophosphate might bind to pyolysin directly to prevent cytolysis. To examine this possibility, pyolysin was incubated with 20 μM farnesyl pyrophosphate or 20 μM geranylgeranyl pyrophosphate for 1 h prior to addition of red blood cells, which are highly sensitive to pyolysin, with 10 HU pyolysin causing >95% hemolysis [5, 31]. Cholesterol was used as a positive control because it is known to bind pyolysin, and, as expected, cholesterol reduced the ability of pyolysin to cause hemolysis (*P* < 0.001, ANOVA; Figure 4B). However, farnesyl pyrophosphate or geranylgeranyl pyrophosphate did not bind directly to pyolysin as there was no significant effect of farnesyl pyrophosphate or geranylgeranyl pyrophosphate on the ability of pyolysin to cause hemolysis (Figure 4B). Together, the data from Figures 3B, 3E, and 4A provide evidence that farnesyl pyrophosphate and geranylgeranyl pyrophosphate increase endometrial cell tolerance to pyolysin.

### Depleting FDPS and GGPS1

**Figure 5.**
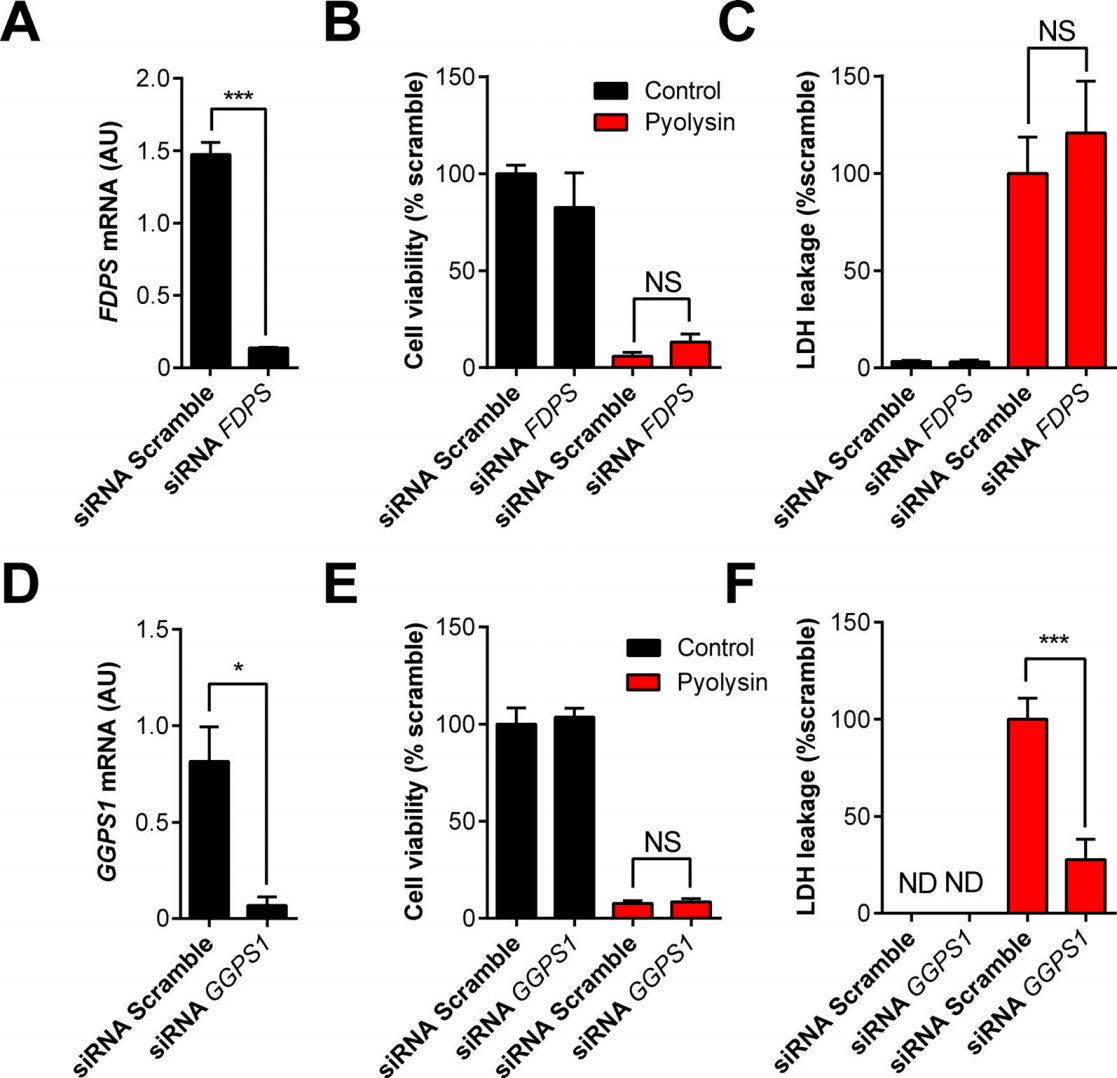
RNA interference of *FDPS* and *GGPS1*. (A–F) Endometrial stromal cells were transfected with scramble siRNA or siRNA targeting *FDPS* or *GGPS1* for 48 h. Cells were incubated for 24 h in serum-free medium and then challenged with control medium black bars (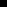) or 100 HU/ml pyolysin red bars (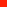) for 2 h. The mRNA expression of each cognate gene was measured by qPCR and normalized to two reference genes, *ACTB* and *RPL19* (A, D). Data are presented as mean (SEM); n = 3 animals. Data were analyzed by Student *t*-test; values differ from scramble, * *P* < 0.05, *** *P* < 0.001. Cell viability was quantified by MTT assay and data expressed as percent of control (B, E), and supernatants were collected to measure the leakage of LDH from cells and data expressed as percent of control challenged with pyolysin (C, F). Data are presented as mean (SEM); n = 5 animals. Data were analyzed by one-way ANOVA and Dunnett multiple comparison post hoc test; values differ from scramble challenged with pyolysin, *** *P* < 0.001, NS = not significant. (Please see the online version for the color figure.)

The synthesis of farnesyl pyrophosphate depends on FDPS (Figure 1). To examine the effect of reducing endogenous farnesyl pyrophosphate, siRNA was used to deplete *FDPS* mRNA expression (Figure 5A). However, depleting *FDPS* did not significantly change cell viability or reduce the leakage of LDH from cells when cells were challenged with pyolysin (Figure 5B and C).

As farnesyl pyrophosphate is converted to geranylgeranyl pyrophosphate by GGPS1 (Figure 1), we wondered if increasing the abundance of endogenous farnesyl pyrophosphate by depleting *GGPS1* mRNA might increase cell tolerance to pyolysin. Using siRNA to deplete *GGPS1* mRNA (Figure 5D) did not affect cell viability (Figure 5E). However, depleting *GGPS1* mRNA reduced the leakage of LDH from cells challenged with pyolysin by 72% (Figure 5F).

Endogenous farnesyl pyrophosphate and geranylgeranyl pyrophosphate abundance can also be increased by inhibiting FDFT1 with zaragozic acid [53]. Treatment of stromal cells with 10 μM zaragozic acid for 48 h increased cell viability when cells were challenged with 100 HU/ml pyolysin for 2 h, compared with untreated cells challenged with pyolysin (36% vs 14% cell viability of control; *P* < 0.05, *t*-test, n = 4). In addition, zaragozic acid also reduced the leakage of LDH into cell supernatants by 50 ± 16% compared with untreated cells challenged with pyolysin (*P* < 0.05, *t*-test, n = 4). Taken together, these data provide further evidence for the importance of the mevalonate-derived isoprenoids in cell tolerance to pyolysin.

### Farnesyl pyrophosphate reduces cellular cholesterol

**Figure 6.**
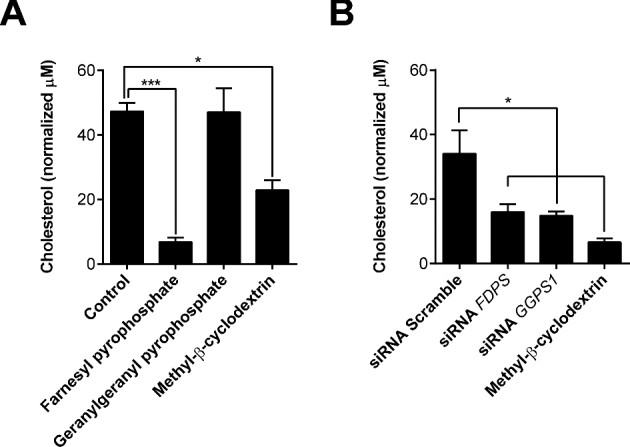
Cellular cholesterol. (A) Endometrial stromal cells were incubated in control serum-free medium, or medium containing 20 μM farnesyl pyrophosphate, 20 μM geranylgeranyl pyrophosphate, or 0.5 mM methyl-β-cyclodextrin for 48 h. (B) Cells were incubated for 48 h in medium containing scramble siRNA or siRNA targeting *FDPS* or *GGPS1*, or cultured with 0.5 mM methyl-β-cyclodextrin as a positive control. Cellular cholesterol was measured and normalized to phospholipid concentrations. Data are presented as mean (SEM); n = 4 independent animals for each experiment. Data were analyzed by one-way ANOVA and Dunnett multiple comparison post hoc test; values differ from control (A) or scramble (B), * *P* < 0.05, *** *P* < 0.001.

We next considered potential mechanisms for farnesyl pyrophosphate increasing cell tolerance to pyolysin. The most obvious mechanism for increasing cell tolerance to cholesterol-dependent cytolysins is to reduce cellular cholesterol, as 90% of cholesterol is in cell plasma membranes [5, 20]. To examine the effect of the mevalonate-derived isoprenoids on cellular cholesterol, endometrial stromal cells were cultured for 48 h with 20 μM farnesyl pyrophosphate or 20 μM geranylgeranyl pyrophosphate, with 0.5 mM methyl-β-cyclodextrin used as a positive control. The cells were collected to measure cellular cholesterol abundance, which was normalized to phospholipid abundance, as described previously [19]. Farnesyl pyrophosphate reduced cellular cholesterol, but geranylgeranyl pyrophosphate had no significant effect (Figure 6A). Depleting *FDPS* mRNA expression using siRNA reduced cellular cholesterol, as might be predicted from the requirement for farnesyl pyrophosphate flux through the mevalonate pathway for cholesterol synthesis. However, depleting *GGPS1* mRNA to increase farnesyl pyrophosphate abundance also reduced cellular cholesterol (Figure 6B). Similarly, cholesterol abundance was reduced by treating cells with 10 μM zaragozic acid for 48 h to increase endogenous farnesyl pyrophosphate abundance, compared with cells in control medium (20.1 ± 1.3 vs 47.3 ± 2.5 μM cholesterol; *P* < 0.001, n = 4, *t*-test). Together, these observations provide evidence for farnesyl pyrophosphate improving cell tolerance to pyolysin by reducing cellular cholesterol.

### NR1H receptors

**Figure 7.**
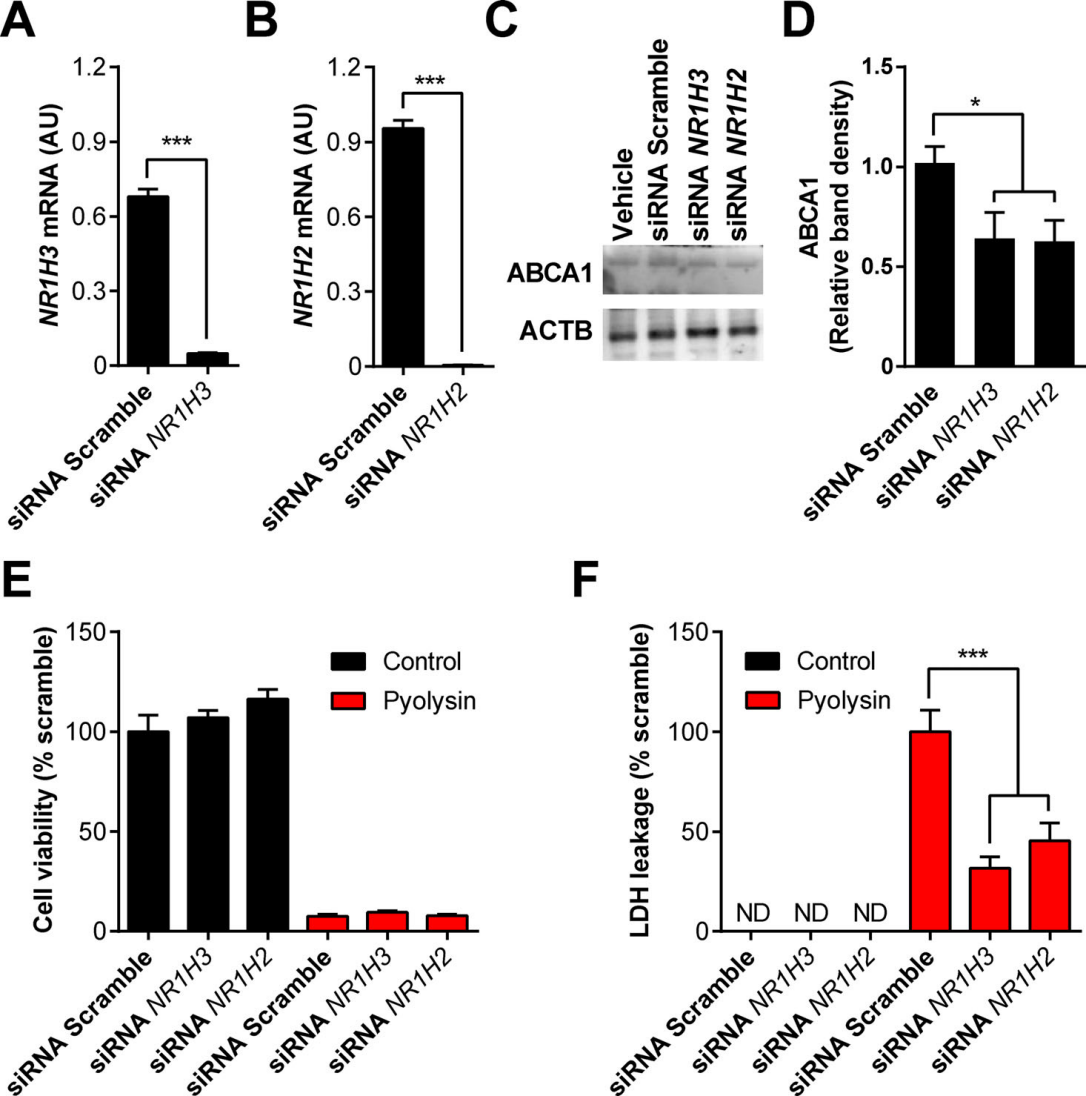
NR1H receptors and cell tolerance. Endometrial stromal cells were transfected with scramble siRNA or siRNA targeting *NR1H2* or *NR1H3* for 48 h. The mRNA expression of each cognate gene was measured by qPCR and normalized to two reference genes, *ACTB* and *RPL19* (A, B). Data are presented as mean (SEM); n = 3 animals. Data were analyzed by Student *t*-test; values differ from scramble, *** *P* < 0.001. (C) Endometrial stromal cells cultured with vehicle or transfected with scrambled siRNA, or siRNA targeting *NR1H3* (si*NR1H3*) or *NR1H2* (si*NR1H2*), were analyzed by western blot for ABCA1 and ACTB abundance. (D) Densitometry data for ABCA1 were normalized to ACTB, and are presented as mean (SEM) from three independent experiments. Data were analyzed by ANOVA and Dunnett pairwise multiple comparison t-test; values differ from scramble, * *P* < 0.05. (E, F) Endometrial stromal cells were transfected with scramble siRNA or siRNA targeting NR1H2 or NR1H3, incubated for 24 h in serum-free medium, and then challenged with control medium black bars (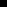) or 100 HU/ml pyolysin red bars (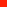) for 2 h. Cell viability was quantified by MTT assay (E), and supernatants were collected to measure leakage of LDH (F). Data are presented as mean (SEM); n = 5 animals. Data were analyzed by one-way ANOVA and Dunnett multiple comparison post-hoc test; values differ from scramble, *** *P* < 0.001. (Please see the online version for the color figure.)

Despite the evidence that farnesyl pyrophosphate increases cell tolerance to pyolysin by reducing cellular cholesterol, how geranylgeranyl pyrophosphate protected cells against pyolysin was unclear, as geranylgeranyl pyrophosphate is not converted to farnesyl pyrophosphate [53]. However, geranylgeranyl pyrophosphate inhibits NR1H3 and NR1H2 activity, whilst farnesyl pyrophosphate does not bind NR1H receptors [28, 54]. We therefore examined whether cell tolerance was affected by inhibition of NR1H3 (encoded by the gene *NR1H3*) or NR1H2 (encoded by the gene *NR1H2*). The expression of *NR1H3* and *NR1H2* mRNA was reduced by >90% using siRNA (Figure 7A and B). Reduction in the abundance of ABCA1 protein further confirmed the effectiveness of the siRNA (Figure 7C and D; Supplemental Figure S1). The reduced expression of *NR1H3* and *NR1H2* mRNA did not significantly increase cell tolerance to pyolysin (Figure 7E), but did reduce the leakage of LDH from cells challenged with pyolysin by 68% and 55%, respectively (Figure 7F). Taken together, these data provide evidence that inhibiting NR1H receptor expression increases cell tolerance to pyolysin.

## Discussion

Here we explored bovine endometrial stromal cell tolerance to the cholesterol-dependent cytolysin pyolysin. As well as addressing a biologically relevant problem for reproduction, the pyolysin dose-dependent sensitivity of stromal cells provides a useful model to examine cell-intrinsic pathways that modulate cell tolerance to pathogens or their virulence factors. Endometrial stromal cell tolerance was dependent on the cholesterol synthesis pathway, but not the MAPK, cell cycle, or metabolic pathways that we screened. In particular, cell tolerance to pyolysin was increased by the mevalonate-derived isoprenoids farnesyl pyrophosphate and geranylgeranyl pyrophosphate. Reducing cellular cholesterol is known to increase cell tolerance to cholesterol-dependent cytolysins, and farnesyl pyrophosphate, but not geranylgeranyl pyrophosphate, reduced cellular cholesterol. However, geranylgeranyl pyrophosphate inhibits NR1H receptors, and using siRNA to target *NR1H3* and *NR1H2* increased cell tolerance to pyolysin. Together the results provide evidence that isoprenoids from the mevalonate pathway increase cell tolerance to pyolysin, and this was associated with reducing cellular cholesterol and inhibiting NR1H receptors.

In our initial screen for cell pathways that could protect against pyolysin, there was no significant effect of manipulating the MAPK, cell cycle, or metabolic pathways. However, manipulating the cholesterol synthesis pathway with atorvastatin or zaragozic acid markedly improved cell tolerance to pyolysin. Atorvastatin is a prototypical statin that inhibits HMGCR to reduce cellular cholesterol [55]. The protective role of atorvastatin in the present study supports previous findings that 1 μM atorvastatin protects bovine endometrial stromal cells against pyolysin by reducing cellular cholesterol [19]. Similarly, 1 μM simvastatin, which is another commonly used statin, protects human airway cells against the cholesterol-dependent cytolysin pneumolysin by reducing cellular cholesterol [56]. The increased cell tolerance to pyolysin in cells treated with zaragozic acid agrees with previous findings that inhibiting FDFT1 increases bovine endometrial stromal cell tolerance to pyolysin in a zaragozic acid concentration-dependent manner [19]. Similarly, inhibiting FDFT1 with YM-53601 increased human airway cell tolerance to pneumolysin, as determined by leakage of ATP from the cytosol [56]. However, in both these studies the protective effects of inhibiting the cholesterol synthesis pathway were not solely attributable to reducing cellular cholesterol [19, 56]. Whilst inhibiting FDFT1 reduces the conversion of farnesyl pyrophosphate to squalene, zaragozic acid treatment of Hep G2 cells also yields farnesyl pyrophosphate and geranylgeranyl pyrophosphate [46]. So, we reasoned that an alternative mechanism for the increase in cell tolerance to pyolysin might be increased abundance of these mevalonate-derived isoprenoids. Unfortunately, measuring the concentration of isoprenoids inside cells was not practicable because isoprenoids are rapidly metabolized, and measurements using mass spectrometry are technically challenging. Furthermore, cell viability after a 24-h challenge with pyolysin might reflect cell repair or replication, as well as tolerance to pyolysin. We sought to more specifically examine cell tolerance using a brief 2-h challenge with pyolysin. Challenging bovine endometrial stromal cells with pyolysin reduced cell viability and increased the leakage of LDH in a concentration-dependent manner to provide a tractable model to explore cell-intrinsic pathways affecting tolerance.

To examine the role of the cholesterol synthesis pathway in cell tolerance, we supplied cells with pathway intermediates (Figure 1). Mevalonate or squalene did not affect cell tolerance to pyolysin, but tolerance was increased by lanosterol, farnesyl pyrophosphate, and geranylgeranyl pyrophosphate. Supplying either farnesyl pyrophosphate or geranylgeranyl pyrophosphate reduced cytolysis and the leakage of LDH from cells challenged with pyolysin. However, reducing the supply of endogenous farnesyl pyrophosphate in cells using siRNA targeting *FDPS*, or using the bisphosphonates etidronate or alendronate to inhibit FDPS, had no significant effect on cell tolerance to pyolysin [45]. Conversely, increasing endogenous farnesyl pyrophosphate, using siRNA to target *GGPS1* to prevent conversion of farnesyl pyrophosphate to geranylgeranyl pyrophosphate, reduced LDH leakage from cells challenged with pyolysin. Similarly, increasing endogenous farnesyl pyrophosphate and geranylgeranyl pyrophosphate by inhibiting FDFT1 with zaragozic acid increased cell tolerance and reduced LDH leakage by half. Together these results provide evidence that approaches to increase farnesyl pyrophosphate or geranylgeranyl pyrophosphate abundance also increased cell tolerance to pyolysin.

Reducing cellular cholesterol is the most obvious mechanism by which altering isoprenoid abundance might increase cell tolerance to cholesterol-dependent cytolysins. Farnesyl pyrophosphate reduces cellular cholesterol in rat hepatocytes [55]. In the present study, supplying endometrial stromal cells with farnesyl pyrophosphate also reduced cholesterol. Furthermore, cholesterol was reduced by increasing endogenous farnesyl pyrophosphate using zaragozic acid to inhibit FDFT1, although inhibiting FDFT1 also reduces the synthesis of cholesterol by reducing the availability of squalene [46]. Similarly, siRNA targeting *GGPS1* to prevent conversion of farnesyl pyrophosphate to geranylgeranyl pyrophosphate also reduced cellular cholesterol. From these results, we suggest that farnesyl pyrophosphate increases cell tolerance to pyolysin by reducing cellular cholesterol.

As supplying exogenous geranylgeranyl pyrophosphate had no effect on cellular cholesterol abundance, we considered an NR1H receptor-dependent mechanism for the action of geranylgeranyl pyrophosphate on cell tolerance. Although the endogenous agonists for NR1H receptors are oxysterols, geranylgeranyl pyrophosphate also inhibits NR1H3 and NR1H2, whereas farnesyl pyrophosphate does not [28, 54]. We reasoned that if geranylgeranyl pyrophosphate inhibits NR1H3 or NR1H2, then siRNA targeting their cognate genes *NR1H3* and *NR1H2*, respectively, might increase cell tolerance. Although siRNA targeting *NR1H3* or *NR1H2* had no significant effect on cell viability, depleting *NR1H3* or *NR1H2* mRNA expression reduced the leakage of LDH from cells challenged with pyolysin by 68% and 55%, respectively. These data provide evidence for an unexpected link between NR1H receptor inhibition and cell tolerance.

Future work might explore which NR1H genes and pathways are linked to cell tolerance. Both NR1H3 and NR1H2 are constitutively active and have a wide range of target genes [28, 29]. Usually, activation of NR1H receptors leads to cholesterol efflux from cells, and in the present study, siRNA targeting *NR1H3* and *NR1H2* reduced the abundance of the ABCA1 cholesterol efflux protein. So our finding that inhibition of NR1H receptors increases cell tolerance to pyolysin was surprising. However, NR1H receptors may also control the distribution of cholesterol in cells, and the susceptibility to pore-forming toxins may depend on the architecture of cholesterol-rich areas in plasma membranes [57]. Additional genes controlled by NR1H receptors that may also be important for cell tolerance to pore-forming toxins include genes associated with regulating inflammation, lipid metabolism, and carbohydrate metabolism [25]. Mevalonate-derived isoprenoids might also alter cell tolerance by affecting the abundance of coenzyme Q10 or dolichols, or protein farnesylation or geranylation although depletion of the isoprenoids did not increase cell sensitivity to pyolysin. It would be interesting to determine the effect of isoprenoids on the number and distribution of pores in the plasma membrane. However, this is not currently tractable in living cells because pyolysin pores are <50 nm diameter, and protrude <7 nm above the membrane surface, as determined by atomic force microscopy and neutron reflectometry in artificial lipid membranes [48]. Similarly, we are aware that our endometrial cells are isolated from normal animals, rather than postpartum animals where other aspects of the postpartum period could influence how cells respond to pyolysin and isoprenoids. Irrespective of the underlying mechanisms, the translation of our findings might be possible by manipulating the cholesterol synthesis pathway in vivo. There is now a need for in vivo experiments, perhaps using intrauterine infusion of an FDFT1 inhibitor to increase farnesyl pyrophosphate and geranylgeranyl pyrophosphate, to confirm our present data and examine if isoprenoids can increase endometrial tolerance to pyolysin, in order to prevent postpartum uterine disease, such as metritis and endometritis.

In conclusion, farnesyl pyrophosphate and geranylgeranyl pyrophosphate increased stromal cell tolerance to pyolysin. Increasing cell tolerance using approaches to increase farnesyl pyrophosphate was associated with reduced cellular cholesterol abundance. Surprisingly, geranylgeranyl pyrophosphate increased cell tolerance to pyolysin independently of cellular cholesterol abundance. However, geranylgeranyl pyrophosphate is an inhibitor for NR1H receptors, and reducing the expression of the *NR1H3* and *NR1H2* genes increased stromal cell tolerance to pyolysin. We suggest that mevalonate-derived isoprenoids increase bovine endometrial stromal cell tolerance to pyolysin by reducing cellular cholesterol and inhibiting the NR1H receptors.

## Supplementary data

**Supplemental Figure S1**. Western blot for ABCA1. Endometrial stromal cells cultured with vehicle or transfected with scrambled siRNA, or siRNA targeting NR1H3 (siNR1H3) or NR1H2 (siNR1H2), were analyzed by western blot for ABCA1 and ACTB abundance. The images of the blots for ABCA1 and ACTB are presented for the three independent experiments.

**Supplemental Table S1**. siRNA sequence for target gene knockdown.

**Supplemental Table S2**. Sequence of reference gene and target gene primers.

**Supplemental Table S3**. Details of the antibodies used in the study for western blotting.

Supplemental data

## Notes

Edited by Dr. Haibin Wang, PhD, Xiamen University
