# Supplementary material for: Isoprenoids increase bovine endometrial stromal cell tolerance to the cholesterol-dependent cytolysin from Trueperella pyogenes
Source: Biol Reprod. 2018 Apr 24;99(4):749–60. doi: 10.1093/biolre/ioy099 (PMC6203874; doi:10.1093/biolre/ioy099)
Supplement: Supplemental data [file ioy099_supp.pdf]

Supplemental Data

Isoprenoids increase bovine endometrial stromal cell tolerance to the cholesterol-dependent cytolysin from Trueperella pyogenes

Sholeem Griffin[1] Gareth D. Healey[1], I. Martin Sheldon[1]

[1] Swansea University Medical School, Swansea University, Swansea, United Kingdom

Supplemental Table S1. siRNA sequence for target gene knockdown.

Supplemental Table S2. Sequence of reference gene and target gene primers.

Supplemental Table S3. Details of the antibodies used in the study for Western blotting.

Supplemental Figure S1. Western blot for ABCA1.

Supplemental Table S1. siRNA sequence for target gene knockdown.

**Gene**	**Direction**	**Sequence (5'→ 3')**
FDPS	Sense	GCACAGACAUCCAGGACAAUU
	Antisense	UUGUCCUGGAUGUCUGUGCUU
GGPS1	Sense	UGACAGAAAUGUUGCAUAAUU
	Antisense	UUAUGCAACAUUUCUGUCAUU
NR1H2	Sense	AGGUGAAGGUGUCCAGUUAUU
	Antisense	UAACUGGACACCUUCACCU
NR1H3	Sense	GCUAAAUGAUGCUGAGUUUUU
	Antisense	AAACUCAGCAUCAUUUAGC

Supplemental Table S2. Sequence of reference gene and target gene primers.

**Gene**	**Direction**	**Sequence (5'→ 3')**
ACTB	Forward	CAGAAGGACTCGTACGTGGG
	Reverse	TTGGCCTTGGGGTTCAGGG
RLP19	Forward	TGTTTTTCCGGCATCGAGCCCG
	Reverse	ATGCCAACTCCCGCCAGCAGAT
FDPS	Forward	ATGACGGGTAAGATCGGCAC
	Reverse	TTCTGCCCATAGTTCTCCTGC
GGPS1	Forward	TATGACCCCTGCCTTCCTGA
	Reverse	TGGTTCTCACTTGTTTACCTGG
FDFT1	Forward	GGCACCCTGAGGAGTTCTAC
	Reverse	GCATACTGCATGGCGCATTT
NR1H2	Forward	TCTTCACCTGGGGGAAAGGA
	Reverse	CTAGGATGACCACAACGGAGG
NR1H3	Forward	GGAGGTACAACCCTGGGAGT
	Reverse	AGCAATGAGCAAGGCAAACT

Supplemental Table S3. Details of the antibodies used in the study for Western blotting.

**Protein target**	**Antigen sequence**	**Name of antibody**	**Manufacturer, catalog #, and RRID**	**Species raised in**	**Dilution used**
**ABCA1**	Recombinant fragment corresponding to Human ABCA1 aa 1800-2260 in UniProtKB O95477 (ABCA1_HUMAN)	Mouse Anti-ABCA1 Monoclonal Antibody, Unconjugated, Clone AB.H10	Abcam, Cambridge, UK Abcam Cat# ab18180, RRID:AB_444302	Mouse monoclonal IgG1 Clone: mAbcam AB.H10	1:500
**ACTB**	Synthetic peptide conjugated to KLH derived from within residues 1-100 of human beta Actin	Anti-beta Actin [mAbcam 8226] antibody ab8226	Abcam PLC, Cambridge, UK Abcam Cat# ab8226, RRID:AB_306371	Mouse monoclonal IgG1 Clone: mAbcam 8226	1:500

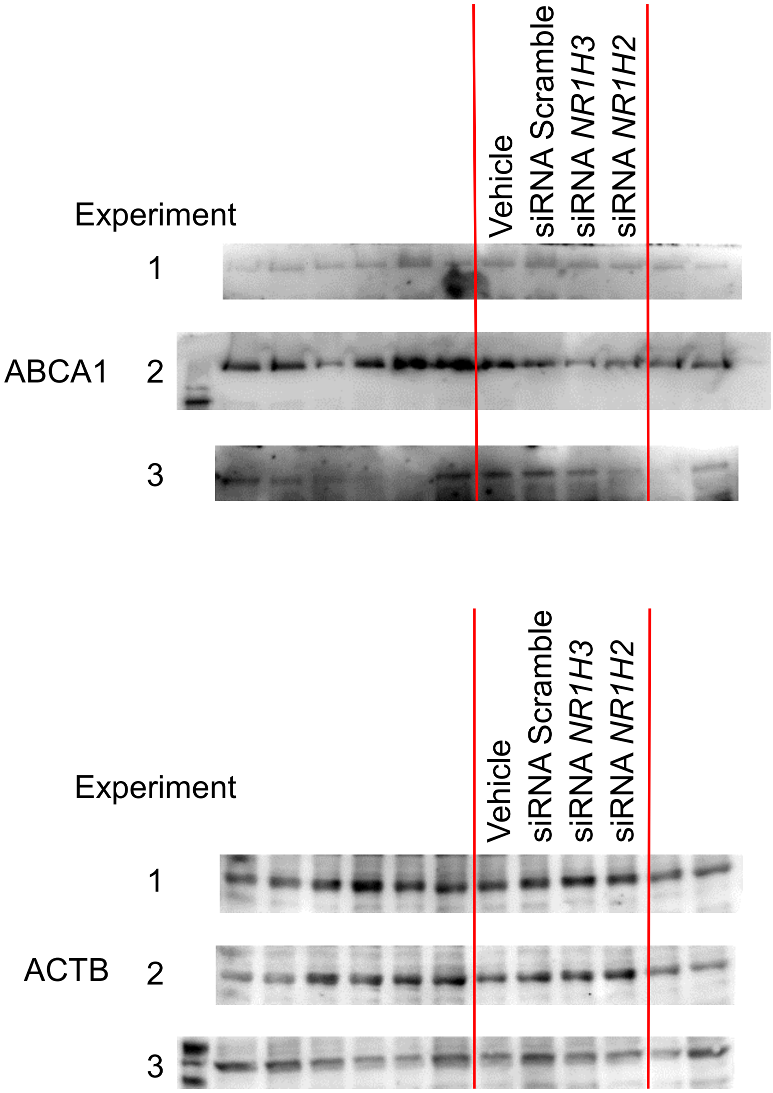

Supplemental Figure S1. Western blot for ABCA1. Endometrial stromal cells cultured with vehicle or transfected with scrambled siRNA, or siRNA targeting NR1H3 (siNR1H3) or NR1H2 (siNR1H2), were analysed by Western blot for ABCA1 and ACTB abundance. The images of the blots for ABCA1 and ACTB are presented for the three independent experiments.
